# Antioxidant and Antiradical Activities of *Hibiscus sabdariffa* L. Extracts Encapsulated in Calcium Alginate Spheres

**DOI:** 10.3390/polym15071740

**Published:** 2023-03-31

**Authors:** Pascal Bevan, Maria Vicenta Pastor, María Pilar Almajano, Idoia Codina-Torrella

**Affiliations:** 1 Chemical Engineering Department, Universitat Politècnica de Catalunya, Av. Diagonal 647, 08028 Barcelona, Spain; 2 Agri-Food Engineering and Biotechnology Department, Universitat Politècnica de Catalunya, Esteve Terrades 8, 08860 Castelldefels, Spain

**Keywords:** spherification, alginate, *Hibiscus sabdariffa* L., encapsulation, oxidation, antiradical

## Abstract

The interest in natural sources with high antioxidant powder has recently increased in several sectors. Ionic gelation methods could be used to protect bioactive substances to control the kinetics and release of these ingredients to the food matrix. This study dealt with the evaluation of the antioxidant capacity and scavenging activity of extracts of *Hibiscus Sabdariffa* L. (HSL) (with 50% ethanol) encapsulated in calcium alginate spheres as a new source for preserving food against oxidative damage. Their antioxidant activity was measured in different o/w emulsions in which HSL spheres reduced the formation of hydroperoxides (~80%) and thiobarbituric-acid-reactive substance products (~20%). The scavenging activity of HSL extracts was measured in different food simulants (water, water acidified with 3% acetic acid, ethanol at 50%, and pure ethanol), and corresponded to 0.20–0.43, 0.31–0.62, and 11.13–23.82 mmol Trolox/mL extract for Trolox equivalent antioxidant capacity (TEAC), 2,2-diphenylpicrylhydrazyl (DPPH), and oxygen radical absorbance capacity (ORAC) assays, respectively. In general, the best antiradical activity was observed in the ethanolic and acidified mediums, in which the highest concentration of released polyphenols ranged from 0.068 to 0.079 mg GAE/mL. This work indicates the potential of alginate spheres for encapsulating antioxidant compounds as an innovative strategy for several industrial applications.

## 1. Introduction

The oxidation of nutritional components (such as proteins, vitamins, minerals, etc.) determines the overall quality of food and directly impacts the consumer’s acceptance of the product. There are different strategies to delay the oxidation of these components during the shelf life of food, of which the incorporation of synthetic and natural antioxidants is one of the most widely used. These substances counteract oxidation by acting through different strategies: as reducing agents, quenchers of radical species, free radical scavengers, or by inactivating prooxidants [1]. Nowadays, the interest in natural sources with high antioxidant power has recently increased in several sectors to replace synthetic antioxidants in industrial applications [2]. Regarding this, the incorporation of plant extracts with a high antioxidant capacity is a current focus of interest for developing new functional products. Apart from that, the economic impact of using these extracts from by-products or under-exploited plant species also has increased the interest in these natural sources [2]. Regrettably, the incorporation of these extracts makes necessary their protection against environmental and processing conditions to preserve their stable properties over time [3]. Different techniques have been proposed, of which encapsulation is one of the most widely used [4]. This technique could use ionic gelation methods, which lead to gel formation [5]. This molecular process protects the bioactive components with a hydrogel formed with specific structural and mechanical characteristics [5]. In general, different polysaccharides and calcium salts are used to obtain these spheres [5,6], which determines their physicochemical characteristics (viscosity, porosity, shape, etc.). Alginate is one of the most widely used polymers, which is used by the industry as a thickener or stabilizer. Alginate is formed by a chain of B-D-mannuronic (M units) acid and alpha-L-guluronic acid (G units) linked with 1–4 glycoside linkage. The gelation of alginate depends on the polymer’s chemical structure (molecular weight and G/M unit ratio, among others) [5]. This polysaccharide has a high affinity to form hydrogels through ionic cross-linking with divalent or trivalent cations, such as Ca^2+^. When calcium ions replace Na^+^ to cross-link different chains of alginate, the network which is formed is commonly described as an “eggbox model”. Two main gelation methods are traditionally used (external and internal gelation). In the internal or indirect gelation process (which has been used in the current study), the gel formation is due to the calcium release into the alginate solution and produces a homogeneous gel structure with pores. The bioactive compounds are mixed with the solution of calcium, and then the mixture is added dropwise into an alginate bath. This method allows better control of the kinetics, and it is commonly used for delivery systems of bioactive compounds in food [5]. A disadvantage of this method of protecting the antioxidant compounds is that encapsulation hydrophilic of low-molecular substances presents an easy diffusion and fast release through the ionic gel network depending on the value of different physicochemical parameters of the medium (pH, temperature, etc.). 

In the literature, different authors have encapsulated plant extracts to protect bioactive compounds against their degradation [3,5,6]. *Hibiscus sabdariffa* L. (HS) (roselle; Malvaceae) is an autogenous plant with around 200 varieties, which is cultivated in different tropical or subtropical areas worldwide [7,8,9]. This edible plant is an important source of dietary nutrients and it is traditionally consumed for its health and nutritional benefits [9]. Frequently, in the food industry, this plant has also been used in herbal beverages and as a flavoring agent [10]. Roselle composition depends on the flowering stage, but also their different varieties, the part of the plant, genetics, environmental conditions, and ecology. Carbohydrates are the main component (considering the whole plant) (~12–54%) [11], followed by protein (3–27%), fat (0.1–13%), and crude fiber (2.3–27%) [11,12]. Moreover, Roselle is an appreciable source of vitamins, minerals, and bioactive components [13], such as the organic acids or phenolic components, which make this edible plant exploitable in the treatment of various chronic diseases (such as cardiovascular disease, cancer, diabetes, or neurological disorders). Their content in phenolic compounds, which are mainly located in the flower’s calyx [14], is principally composed of anthocyanins and flavonoids [12]. De Moura et al. (2018) [6] encapsulated extract from *Hibiscus sabdariffa* L. by the ionic gelation method and evaluated the release of anthocyanins under simulated gastrointestinal conditions. The results of their studies demonstrated that microcapsules protected the bioactive compounds of HS and suggested that this application could be technically feasible. 

The aim of this study was to determine the antioxidant capacity and scavenging activity of extracts of *Hibiscus sabdariffa* L. encapsulated in small calcium alginate spheres as a new potential natural source for preserving food products against oxidative damage. In this context, primary and secondary oxidation activity was measured in o/w emulsions in which these spheres were previously incorporated. The radical scavenging activity of these samples was also evaluated by using different methods (TPC, TEAC, DPPH, and ORAC, in four food simulants (water, water with 3% acetic acid, ethanol 50% and, ethanol 100%). The release of the main polyphenolic bioactive compounds of *Hibiscus sabdariffa* L. extract (chlorogenic acid, quercetin and, kaempferol) during the storage of these samples was also quantified. This study responds to the evaluation of the potential viability of these HSL gelling spheres as new ingredients for industrial applications. 

## 2. Materials and Methods

### 2.1. Materials

#### 2.1.1. Reagents 

All reagents were purchased from Sigma Aldrich (Barcelona, Spain) except for HPLC-MS solvents, which were supplied by Panreac (Barcelona, Spain) and alginate (AlgogelTM 3001, INS 401), which was supplied by Algaia (Paris, France).

#### 2.1.2. Plant Material and Preparation of *Hibiscus sabdariffa* L. Extracts

*Hibiscus sabdariffa* L. plants were provided by a local market in Barcelona. Flowers (from red calyces) were accurately separated from the rest of the plant and dried at ~35 °C for ~1 week, until constant weight. Then, dried flowers were ground with an electric blender (Krups F203, Barcelona, Spain) and sieved. Plant powder (HSP) was then packaged in different amber glass bottles and stored in a desiccator to be preserved from light until the performance of the analyses. 

Plant extracts were obtained with ethanol/water (50%, v/v). In of all them, 9.1 g of HSP was extracted at a 1:10 ratio (powder/solvent, w/v), in a dark flask glass, and was stirred using a multiposition magnetic stirrer (Ovan, MM90E, Barcelona, Spain) for 6 h at room temperature (~20 °C); then, after leaving for 30 min, the supernatant was accurately separated after centrifugation (Orto Alresa Mod. Consul, Ajalvir, Madrid, Spain) at 2500 rpm for 15 min, extracts were vacuum filtered, and, subsequently, the supernatant was accurately collected. All supernatants were put in small vials and stored at −80 °C until performing the corresponding analyses. 

### 2.2. Sphere Preparation and Encapsulation of HSL Extract

**Figure 1:**
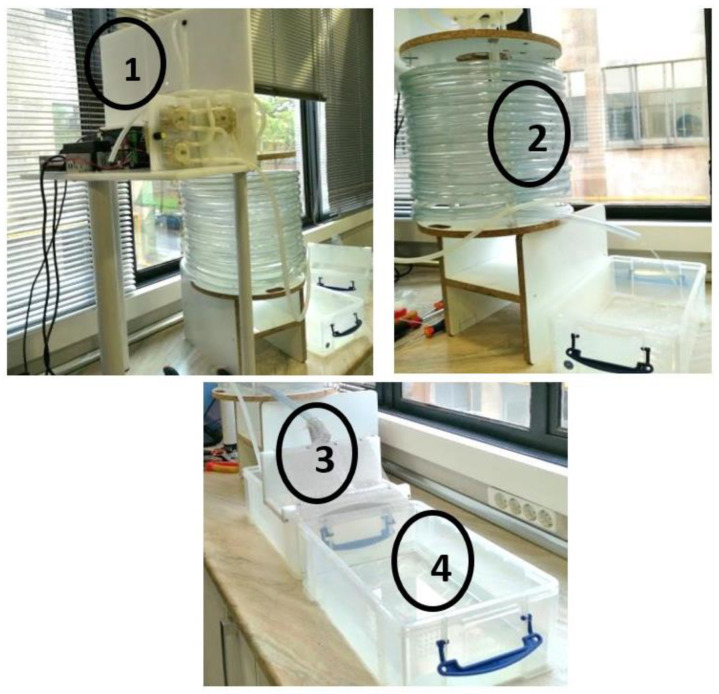
Device for sherification, developed by Mym Group (Barcelona). (**1**) Feeding tube; (**2**) sodium alginate tube (5 m, 3 min); (**3**) collector of sodium alginate solution; and (**4**) bath of distilled water.

In the current study, two types of spheres were prepared: (type 1) spheres with HSL extract in 50% EtOH and (type 2) spheres with ethanol 50% ethanol and 0.1% blue colorant (to make them visible), without extract, as control. The type of sphere used (with or without HSL extract) depended on the objective of the study. A solution of sodium alginate (S1) was firstly prepared (5 g alginate/L of deionized water), which was left at rest overnight, in order to make the air bubbles disappear. The day after, other homogeneous solutions (S2) were prepared, which consisted of 24.8% of each type of initial solution ((1) or (2)), xanthan gum (0.5%, w/v), calcium chloride (0.25%, w/v), saccharose (9.95%, w/v), and the rest was deionized water (64.5%, w/v). The solution that contained the HSL extract (50% EtOH) was then beaded dropwise onto the alginate solution (S1). For this purpose, we used a device designed for Mym Group (Barcelona) (Figure 1). As shown in Figure 1, after absorbing the solution with alginate (S1), this solution was added dropwise in a tube that contained S2 solution, and the gelation of the S1 rapidly occurred. Spheres were finally washed in a distilled water bath, and they were immediately collected and superficially dried through a cellulose filter at room temperature (~20 °C). Spheres with EtOH 50% (EtOH:H_2_O), without HSL extract, were prepared following the same methodology. All spheres were stored at room temperature until use.

### 2.3. Stabilization Studies of Spheres

#### 2.3.1. Physical Characterization of Spheres 

The size, morphology, and color of type 1 and type 2 spheres were determined. Visual morphology and color of spheres were estimated by direct observation. After performing the diffusion assay with HSL spheres (type 1), spheres were carefully separated and superficially dried (filter paper) from their corresponding simulants. The weight and size of spheres were measured over the storage time. To determine the size of spheres, the longest and shortest diameters of ten randomly selected units were determined. The diameter was measured by using a caliper, and it was expressed by averaging the longest and shortest diameters. The weight of spheres was determined by means of ten spheres by using an analytical balance. 

#### 2.3.2. Diffusivity Assay 

A diffusivity assay of antioxidant bioactive components from HSL spheres was performed by using spheres with HSL extract (50% EtOH) and four different food systems (water, water with acetic acid (3%), pure EtOH, and EtOH 50% (EtOH:H_2_O)). This selection was based on the agreement with the Regulation of the European Commission (EU) No.10/2011 on plastic materials and articles intended to come into contact with food. Spheres were immersed with the selected solvent (2 spheres in 1 mL) for a period of time of 72 h and samples were collected every 5 min (the first hour), every hour (until 6 h), and later every 24 h. The food systems (without spheres) were stored at −80 °C until their subsequent analyses. The antiradical activity of solvents was then evaluated according to those exposed in Section 2.3.3. The overall assay was performed in triplicate.

#### 2.3.3. Radical Scavenging Activity

Radical scavenging activity of food simulants was found by determining the total phenolic compound content, Trolox equivalent antioxidant capacity, 2,2-Difenil-1-picrilhidrazil radical scavenging activity, and oxygen radical antioxidant capacity. All analyses were performed in triplicate. 

##### Content of Total Phenolic Compounds

Total phenolic compound (TPC) content was determined by colorimetric spectrophotometry according to the Folin–Ciocalteu (FC) method and considering some modifications [15]. Simulant solvent (7.6%, v/v) was mixed with a FC-reactive (30.8, v/v) sodium carbonate solution at 20% (30.8% v/v) and Milli-Q water (30.8%, v/v). Mixtures were allowed to stand for 1 h at room temperature and preserved from light. The absorbance was measured at 765 nm, at room temperature, with a plate reader UV spectrophotometer (FLUOstar OMEGA, Perkin-Elmer, París, France). TPC was calculated by interpolating the absorbance of the sample against a calibration curve made with a standard solution of gallic acid at fifteen different concentrations (0 to 18 µg/mL) (R^2^ = 0.999). The results were expressed as mg gallic acid equivalents (GAE) per mL of solvent (w.s.).

##### Trolox Equivalent Antioxidant Capacity

The Trolox equivalent antioxidant capacity (TEAC) assay was performed as described by Gallego et al. [16] using a micro-plate reader. Extracts were evaluated by 2,2′-azinobis(3-ethylbenzthiazoline-6-sulfonate) (ABTS) (7 mM). The absorbance of samples was measured by means of a plate reader UV–vis spectrophotometer (FLUOstar OMEGA, Perkin-Elmer, París, France) at 734 nm for 20 min and at 30 °C. TEAC values were obtained according to a calibration curve made with Trolox at eight different concentrations, which ranged from 0.1 to 0.5 mmol Trolox/mL (R^2^ = 0.996). The results were expressed as μmol of Trolox equivalents per mL of extract (w.s.). 

##### 2,2-Difenil-1-Picrilhidrazil (DPPH) Radical Scavenging Activity Assay

The DPPH radical scavenging activity assay was performed according to Villasante et al. (2020) [17]. Solutions were prepared according to 9.1% (v/v) of the extract with 90.9% (v/v) of 2,2-Diphenyl-1-picrylhydrazyl solution. The absorbance was measured by using a UV–vis spectrophotometer (FLUOstar OMEGA, Perkin-Elmer, Paris, France) at 517 nm by using a micro-plate reader, over 75 min, measuring every 15 min. DPPH values were determined according to a calibration curve, which ranged from 0.02 to 0.3 mmol Trolox/mL (R^2^ = 0.993). The results were expressed as mmol of Trolox equivalents per mL of extract (w.s.).

##### Oxygen Radical Antioxidant Capacity (ORAC)

The ORAC method was adapted from [15]. Samples were previously diluted with PBS at the proportion of 1:50. The assay was performed with an automated fluorescence microplate reader equipped with a temperature-controlled incubation chamber (Fluostar Omega, BMG, Ortenberg, Germany) and incubated at 37 °C. In each microplate, samples were added at the concentration corresponding to 18.2% (w/v), and then fluorescein was added at a concentration corresponding to 63.6% (w/v). AAPH (0.3 M) was finally added at the final proportion of 18.2%, and fluorescein was measured at 2-min intervals for 2 h. The ORAC value was calculated using a regression equation relating to Trolox concentration between 20 and 350 mmol Trolox/mL (R^2^ = 0.994). The net area under the fluorescence decay curve in each concentration was also calculated. The results were expressed as mM of Trolox per mL of extract (w.s.). 

#### 2.3.4. Characterization of Phenolic Compounds by HPLC

Three different compounds (chlorogenic acid, quercitin, and kaemferol) were analyzed in the four previously mentioned food systems by HPLC-DAD [14] at the moment in which the maximum antiradical activity was reached (once the diffusivity stabilization occurred, at 6 h of storage). An HPLC system (Spectra-Physics 8810, Thermo Electron Corporation, Asheville, NC, USA) which was equipped with a diode array detector (DAD SP 8490 UV-Visible, Thermo Electron Corporation, Asheville, NC, USA) was used. A volume of 75 μL of the extract was filtered (0.22 μm) and injected into an analytical C18 column (Mediterranean 15 cm, Teknokroma, S.C.L. Spain) at 25 °C. The mobile phase was composed of 0.5% formic acid (v/v) in MilliQ water (eluent A) and 0.5% formic acid in acetonitrile (eluent B). The elution gradient corresponded to 95% A and 5% B, under isocratic conditions. The run time was 25 min and the solvent flow rate corresponded to 0.8 mL/min. The detector wavelength was set at 280 nm and 325 nm in order to quantify the corresponding polyphenols, the concentration of which was calculated using specific calibration curves. The results were expressed as ppm of the corresponding phenolic compound. Characterization of these compounds was performed in triplicate.

### 2.4. Antioxidant Activity of in o/w Emulsions

#### 2.4.1. Oil-in-Water Emulsion Preparation

**Figure 2:**
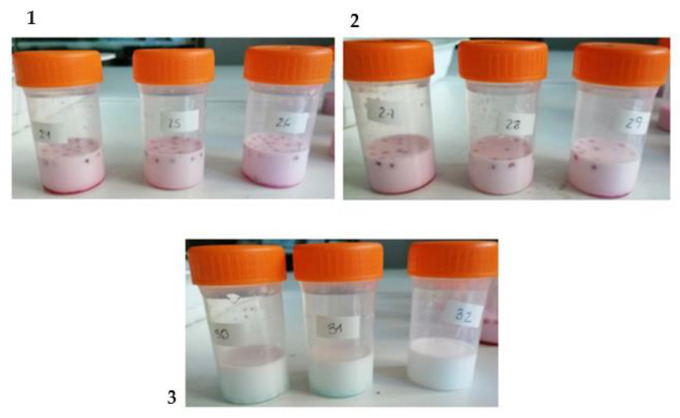
Oil-in-water emulsions with different type of spheres. (**1**) Emulsions with 20 spheres of HSL extract (HSL20), (**2**) emulsions with 10 spheres of HSL extract (HSL10), and (**3**) emulsions with 20 spheres of 50% ethanol (EtOH). Note: HSL: *Hibiscus sabdariffa* L.

Oil-in-water (o/w) emulsion preparation was as follows: Tween-20 (1%, w/w), Milli-Q water (89%, w/w), and methyl linoleate (MeLo, 10%, w/w) oil were used. Tween-20 and Milli-Q water were previously diluted by using a magnetic stirrer to disperse them. In this mixture, the MeLo oil was added dropwise with continuous sonication, for 10 min, using an ultrasonic homogenizer (Hielscher, UP200S, Teltow, Germany) in an ice bath. In order to evaluate the antioxidant capacity of HSL extract on the emulsions, two different batches of samples were prepared. Spheres (10 or 20 units) (sphere type 1, see Section 2.2) were introduced into their respective plastic containers, and then, 10 g of the emulsion was carefully incorporated (Figure 2). All plastic containers were then allowed to oxidize in an oven, at the same time, and were incubated at 33 °C for 10 days in darkness and with constant slow agitation. A third batch of samples (control samples, sphere type 2, see Section 2.2) was also prepared, in which 20 units of spheres with 50% ethanol were added to 10 g of the emulsion, following the same procedure.

#### 2.4.2. Primary Oxidation Measures (Peroxide Value)

The primary oxidation of emulsions was determined by the peroxide value (PV) method, using ferric cyanide [17]. Emulsion drops (of ~19–25 mg of weight) were mixed with 1 mL of absolute EtOH, and the mixture was then completely homogenized with a vortex. In a plastic cuvette, a specific volume of this solution was a mixture with the necessary amount of absolute EtOH to have a final volume of 4 mL, and then 1.8% (v/v) FeCl_2_ (in 37% HCl) and 1.8% (v/v) of ammonium thiocyanate were added. The blank contained 4 mL of absolute EtOH and 1.8% (v/v) of each reactant. PV values were determined from a calibration curve obtained with the official method to determine the hydroperoxides in pure oil (R² = 0.998). The absorbance measure was determined during storage time (10 days), each day, by using a UV–vis spectrophotometer (Zuzi spectrophotometer 4201/20, Auxilab, Navarra, Spain) at 500 nm, and the results are expressed as milli-equivalents of hydroperoxides per kilogram of emulsion (meq hydroperoxides/kg emulsion). Measures were taken in triplicate.

#### 2.4.3. Secondary Oxidation Reactions (Thiobarbituric Acid Reactive Substances, TBARS)

Malondialdehyde (MDA) content was determined according to [18]. A percentage of 8.3% (v/v) of each emulsion was mixed with 8.3% (v/v) of BHA and 83.4% (v/v) of thiobarbituric acid (TBA) solution. Samples were then incubated in a water bath (which was at 95 °C) for 10 min, and then, they were centrifuged (Orto Alresa Mod. Consul, Ajlvir, Madrid, Spain) at 2500 rpm for 5 min. The supernatant of each tube was accurately removed, and the absorbance of this fraction was measured at 531 nm by using a UV–vis spectrophotometer (Zuzi spectrophotometer 4201/20, Auxilab, Navarra, Spain). Results were expressed as milligrams of malondialdehyde per kilogram of emulsion (mg MDA/kg emulsion). Measures were taken in triplicate.

#### 2.4.4. pH Value

Measures of pH were obtained by using a pH meter (GLP21, Criston Instruments, Barcelona, Spain). Measures were performed in triplicate.

### 2.5. Statistical Analysis

The overall study was performed in triplicate. Statistical analysis was performed with MINITAB 17 software (Minitab, Inc., State College, PA, USA). Data were analyzed using one-way analysis of variance (ANOVA), and Tukey’s multiple comparison test was used to determine significant differences between samples, with a value of *p* < 0.05 being considered significant.

## 3. Results and Discussion

### 3.1. Morphology, Size and Color of HSL Spheres

**Figure 3:**
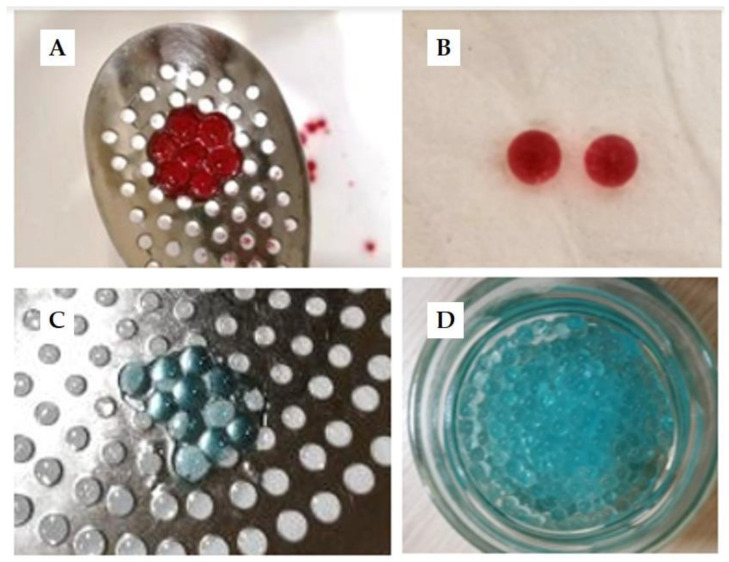
Morphology and size of alginate spheres obtained with (**A**,**B**) HSL extract (50% EtOH) and (**C**,**D**) with blue colorant. Note: HSL: *Hibiscus sabdariffa* L.

Figure 3 shows the images of the spheres obtained in this study, produced with and without HSL extract (50% EtOH). The diameter of all spheres ranged from 2 to 3 mm. No differences were observed among spheres with HSL extract and spheres without the extract. The color of spheres depended on the pigments contained in their corresponding compositions. Spheres elaborated with HSL extract (Figure 3A,B) were red-colored due to their content of the most important pigment of this plant, the anthocyanins [13]. On the contrary, spheres produced only 50% EtOH (Figure 3C,D) were blue-colored because of the blue colorant which was incorporated in their formula to improve their visibility.

As observed in Figure 3, the spheres obtained were morphologically similar, and they presented a homogeneous spherical shape. The high viscosity of the alginate solution probably explains why these particles became spherical. It has been described that the gelation process can be impacted by the concentration of alginate solution [4,19] such that a higher concentration of this biopolymer (1–4%, w/w) molecules increases the number of cross-linking interactions with the bivalent ions and reinforces the structure of the matrix. Similar results were obtained by other authors. Machado et al. (2022) [19] studied the encapsulation of phenolic extracts from spirulina sp. with alginate and reported spheres became more spherical at higher concentrations of sodium alginate. De Moura et al. (2019) [6] also encapsulated bioactive compounds from Hibiscus extract by internal gelation and obtained similar particle-sized spheres (~2 mm). Other physicochemical parameters also impact the gel formation process and determine the characteristics of the sphere, such as the concentration of the calcium ions, the characteristics of the alginate (molecular weight or ratio of M/G units), the processing parameters (pH, time of contact the temperature of processing), and the composition of alginate gels (mixtures of alginate with other polymers or ingredients) [5].

### 3.2. Difussivity Assay and Scavenging Activity

**Figure 4:**
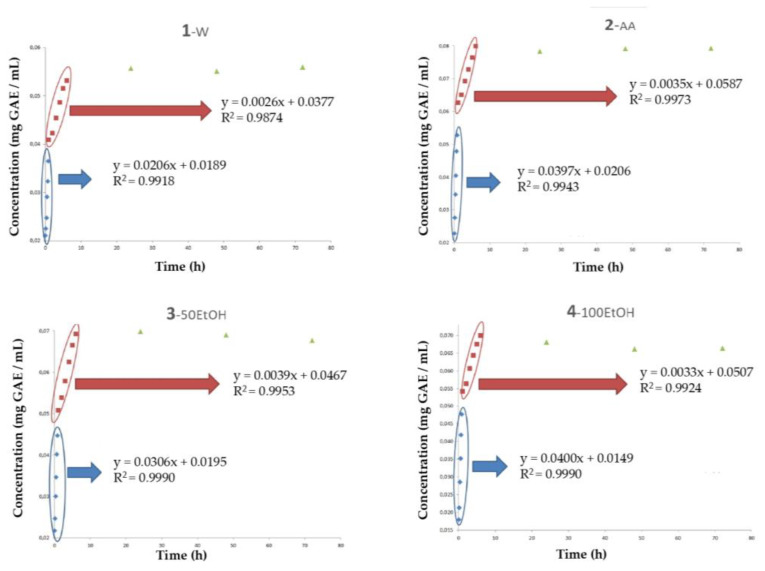
Evolution of total phenolic compounds (TPC) of samples during a storage time of 72 h. Simulants: (**1**) W: water medium, (**2**) AA: acidified water with 3% aceic acid (v/v), (**3**) 50EtOH: ethanol/water 50:50 (v/v), (**4**) 100EtOH: pure ethanol; Concentration: mg gallic acid equivalents (GAE) per mL of extract (w.s.). Time: hours. HSL: *Hibiscus sabdariffa* L.

The release of total phenolic compounds from the alginate spheres with HSL extract was evaluated in different mediums: water (W), acidified water with 3% acetic acid (v/v) (AA), pure ethanol (100EtOH), and EtOH 50% (v/v) (50EtOH). Different mediums were set with the objective of simulating some of the most representative food systems. Figure 4 shows the release behavior of TPC in spheres contained in these simulants. As observed, the maximum release of the polyphenolic compounds occurred during the first 6 h. From that moment, this content remained similar in all samples. In the W medium, the maximum value of TPC corresponded to 0.056 mg GAE/mL, which was lower (*p* < 0.05) than the one observed in the other samples. Simulants with ethanol (100EtOH and 50EtOH) presented a similar released TPC content (*p* > 0.05), which corresponded to 0.068 and 0.070 mg GAE/mL, respectively. On the contrary, the AA matrix presented the highest content in TPC (0.080 mg GAE/mL) (*p* < 0.05), which demonstrated that the low pH of the medium improved the release of these bioactive substances from the gelling structure. The diffusion of the polyphenolic substances from the sphere to the external medium could be explained by different release mechanisms, which are highly affected by environmental conditions. Among others, the pH determines the percentage of release in substances encapsulated by alginate gels, which is attributed to the behavior in solubility of the alginate constituents (β-D-mannuronic and α-L-guluronic acids) at different pH values. When the pH value of the external medium is above the pKa value of both acids (3.38 and 3.65, for β-D-mannuronic and α-L-guluronic acids, respectively), the release of internal compounds occurs because of the increase in solubility of the polymeric gel [20].

Concerning the regression results of TPC release in these four mediums (Figure 4), all samples presented a model which was characterized by a calibration line with three different sections: a first section that ranged from 0 to 50 min, a second section that ranged from 50 min to 6 h, and a last section that ranged from 6 to 72 h. In all samples, the first stage was characterized by the highest velocity of the diffusivity phenomena. Data obtained from the regression equations showed that the velocity of the release of TPC (slope of the line) corresponded to water (0.020) < EtOH50% (0.030) < pure ethanol (0.040) ≈ acid water (0.040). The efficiency of ethanol solvents for the selective extraction of polyphenols probably could explain these results. In the second part of the regression line (from 50 min to 6 h), the diffusivity of TPC decreased by ~90% in all mediums, probably because of the saturation of the external medium (Table 1). Finally, from 6 to 72 h of storage (third step), the diffusion of total polyphenols remained constant in all samples, which demonstrated that from 6 h onwards, no further diffusion of compounds occurred. Luong et al. [21] reported similar results in tea (*Camellia chrysantha*) extracts encapsulated in alginate and chitosan nanoparticles. These authors studied the release kinetics of polyphenols from above these nanoparticles depending on the pH of the solution and the different compositions of the hydrogel membrane. In all cases, during the first hour, 50% of total polyphenols were released. A continuous release stage was described during the following 10 h, in which over 90% of total polyphenol content was released from those nanoparticles. From 10 to 30 h, TPC release practically remained stable. These authors also described that TPC released from the nanoparticles in the acidic environment was lower than that observed in neutral solutions. Koksal E. et al. [22] studied the controlled release of vitamin U (S-mehylmethionine) from microencapsulated Brassica oleracea L. extracts with gelatin/sodium alginate (3:5:1) polymers at different pH values. These authors also reported that during the first hour, 50% of total vitamin U was released. However, in that case, it took practically 24 h to reach the maximum value, which indicates the slow release of vitamin U, probably due to the composition of the hydrogel. Maximum release was observed at neutral pH, whereas at acid pH, the diffusion values corresponded to about one-fifth of that value.

**Figure 5:**
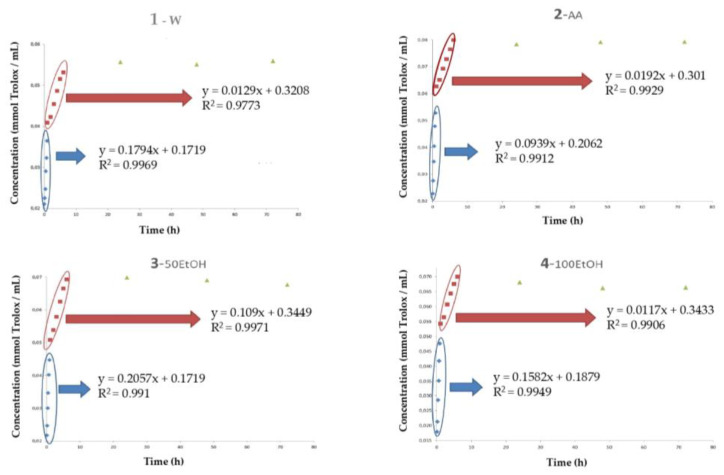
Evolution of Trolox equivalent antioxidant capacity (TEAC) of samples during a storage time of 72 h. Simulants: (**1**) W: water medium, (**2**) AA: acidified water with 3% acetic acid (v/v), (**3**) 50EtOH: ethanol/water 50:50 (v/v), (**4**) 100EtOH: pure ethanol; Concentration: mmol Trolox per mL of extract (w.s.); Time: hours; HSL: *Hibiscus sabdariffa* L.

**Figure 6:**
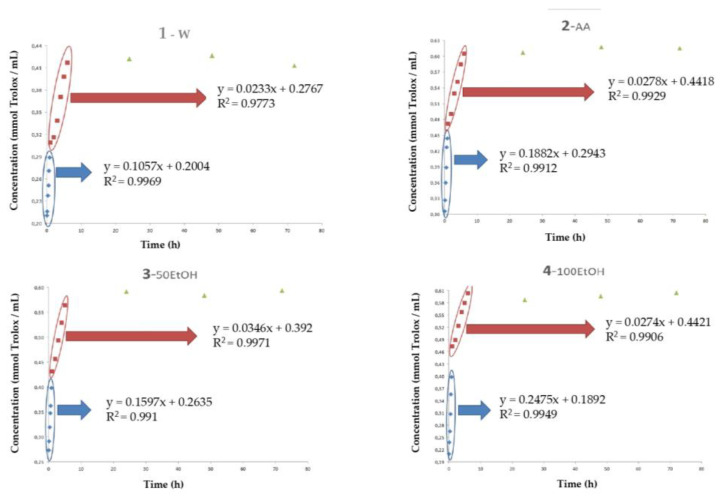
Evolution of 2,2-difenil-1-picrilhidracilo (DPPH) of samples during a storage time of 72 h. Simulants: (**1**) W: water medium, (**2**) AA: acidified water with 3% aceic acid (v/v), (**3**) 50EtOH: ethanol/water 50:50 (v/v), (**4**) 100EtOH: pure ethanol; Concentration: mmol Trolox per mL of extract (w.s.); Time: hours; HSL: *Hibiscus sabdariffa* L.

All samples were also evaluated for their scavenging activities by DPPH free radical scavenging, TEAC, and ORAC assays. The results obtained were in accordance with those observed in diffused TPC. Although TEAC and DPPH methods are similar (both evaluate the ability of the antioxidants to neutralize free radicals, using the radical cation ABTS^•+^ in the TEAC method and the radical DPPH^•^ in the DPH method), some differences were observed among samples. Figure 5 and Figure 6 show the evolution of oxidative stability (TEAC and DPPH assays) of all simulants during 75 h of storage. Concerning the TEAC method (Figure 5), along the first section, the slope obtained in AA sample was ~50% lower than the observed in the other simulants, which indicated that in this medium existed a lower antiradical capacity to inhibit the ABTS^•+^ radicals. On the contrary, the simulant that presented the best antiradical capacity corresponded to 50EtOH. From 50 min to 6 h of storage, the AA simulant showed the best radical scavenging capacity. In Section 3, it was observed that no differences were observed among these samples at the end of the storage time (Table 2). A hypothesis that could explain these results in the acidified medium is that they are not due so much to the antiradical capacity as to the fact that the acidic medium prevents an adequate development of the method.

Regarding the DPPH method, during the first section of the regression line, results obtained showed that the 100EtOH simulant presented the highest slope if compared with the other simulants, which could be attributed to the highest antiradical efficiency of this sample with DPPH^•^ radical. In the second section, simulants that presented the best antiradical capacity corresponded to 50EtOH, followed by AA and 100EtOH simulants, which exhibited similar results (Figure 6). As observed in Figure 6 and Table 3, W simulant exhibited the worst antiradical capacity. Concentration values of DPPH reached once the radical scavenging products in the simulants were saturated demonstrated that the released in water was lower and, for this reason, the W simulant exhibited the worst antiradical capacity. While, on the contrary, the AA, 50EtOH, and 100EtOH simulants exhibited similar results (*p* < 0.05). These samples reached concentrations of Trolox ~ 31% higher than those observed in W.

**Figure 7:**
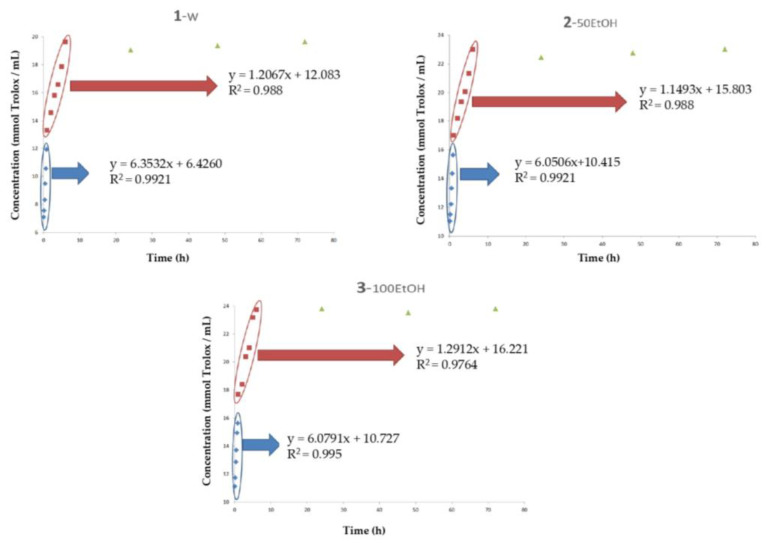
Evolution of the oxygen radical absorption capacity (ORAC) of samples during a storage time of 72 h. Simulants: (**1**) W: water medium, (**2**) AA: acidified water with 3% acetic acid (v/v), (**3**) 100EtOH: pure ethanol, 50EtOH: ethanol/water 50:50 (v/v); Concentration: mmol Trolox per mL of extract (w.s.); Time: hours; HSL: *Hibiscus sabdariffa* L.

The ORAC assay measures the global capacity of antioxidants to neutralize peroxide radicals. In this case, since this test is highly sensitive to pH, the ORAC method was not carried out on the samples that contained acidified water. Results obtained with this method also presented some differences in the sections of the regression model. Concerning the first section, the solvent that presented the best scavenging activity corresponded to the W, followed by 50EtOH and 100EtOH (Figure 7 and Table 4). Constrariously, in the second and third sections, 100EtOH medium exhibited the best results.

Considering the overall results, the anti-radical capacity of the simulant that contained ethanol exhibited the best results. On the contrary, the medium with water was presented as the worst. Polyphenolic compounds seem to have better antiradical activity in polar mediums because of their possible dissociation [15]. Alkaline conditions also may enhance their power as radical scavengers. The best scavenging activity was observed in all simulants after 6 h of storage, which remained constant in all samples until the end of their storage time, probably because of the stabilization in the diffusion of TPC after 6 h.

In order to calculate the migration through the hydrogel of the individual compounds, the concentration of three individual compounds (chlorogenic acid, quercetin, and kaempferol) in the simulant was determined by HPLC-DAD. The moment in which, based on the results obtained previously, the maximum had been reached, close to saturation, was chosen. As observed (Table 5), chlorogenic acid was the most abundant phenolic compound (initial HSL extract and in all simulants), followed by quercetin and kaempferol. In the literature, it has been described that more than 95 phenolic compounds have been identified in this plant, of which quercetin, kaempferol, and chlorogenic acid are some of the most frequently reported [6,10,23]. Among others, the presence of these substances has been strongly correlated with the nutraceutical value and medical properties of these extracts [12,24]. Spheres immersed in 100EtOH simulant exhibited the highest release of chlorogenic acid (*p* < 0.05), probably because of the best extraction affinity of phenolic acids for ethanol [23]. Similar results were observed in the chlorogenic acid release in W and AA mediums (Table 5). Obouayeba et al. (2013) [25] also observed the major extraction yields of flavonoids of HSL in mediums with methanol. On the contrary, the amounts of quercetin and kaempferol increased in spheres immersed in water, probably because of the high solubility of both components in this medium. The results showed that quercetin and kaempferol losses decreased (*p* < 0.05) according to W > AA > 100EtOH > EtOH:W.

### 3.3. Changes of Diameter and Weight of HSL Spheres Contained in Different Simulants with Storage

**Figure 8:**
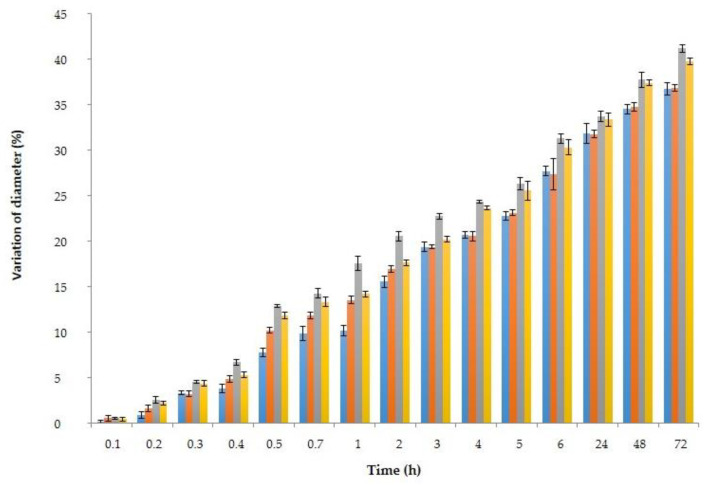
Variation of diameter (%) of spheres with HSL extract during their storage. Water (●), acidified water 3% (●), ethanol 50% (●), and ethanol 100% (●). Values expressed corresponded to the average values and vertical bats to their corresponding standard deviation (n = 3).

**Figure 9:**
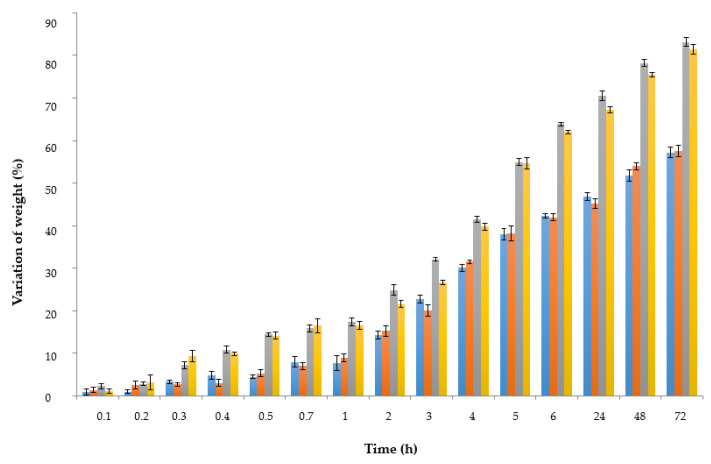
Variation of weight (%) of spheres with HSL during their storage. Water (●), acidified water 3% (●), ethanol 50% (●), and ethanol 100% (●). Values expressed corresponded to the average values and vertical bats to their corresponding standard deviation (n = 3).

The percentage of variation in diameter and weight of spheres is shown in Figure 8 and Figure 9. As observed, variation of both parameters depended on the time that spheres remained in contact with each simulant, and also on the composition of the simulant (water, acidified water, ethanol at 50%, or pure ethanol).

As observed in Figure 8 and Figure 9, the highest differences in variation of weight of spheres corresponded to those particles contained in mediums with ethanol (50EtOH and 100EtOH), probably because of the higher volatility of ethanol in comparison with the other solvents. Spheres immersed in 100EtOH medium showed the biggest changes. On the contrary, spheres immersed in water and acidified water did not present significant (*p* < 0.05) differences if comparing them. Although from 6 to 72 h the diameter and the weight of spheres were still changing, no diffusion of their internal liquid was observed in any simulant.

### 3.4. Antioxidant Activity of Spheres in o/w Emulsions

**Figure 10:**
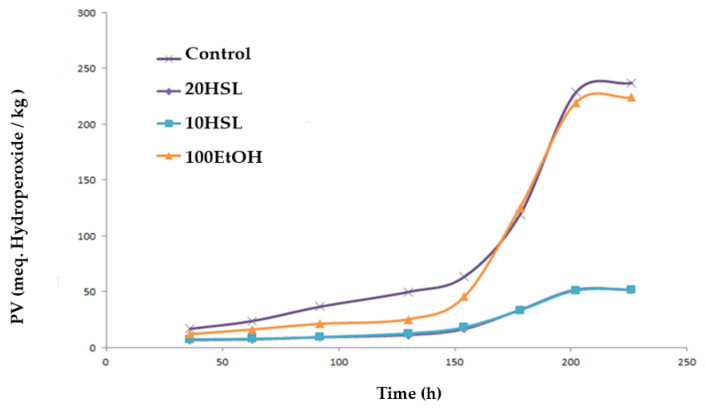
Evolution of peroxide value of emulsions during their storage at 33 °C. Control: emulsion without anything; 100EtOH: emulsion with spheres of pure 50% ethanol; 10HSL: emulsions with 10 spheres of HSL extract; 20HSL: emulsions with 20 spheres of HSL extract. HSL: *Hibiscus sabdariffa* L. Emulsion: Tween-20 (1%, w/w), Milli-Q water (89%, w/w) and methyl linoleate (MeLo, 10%, w/w) oil. PV: Peroxide value, meq. Hydroperoxide/kg emulsion.

The evaluation of primary lipid oxidative reactions was carried out through a peroxide value assay, which was performed during a storage time of 240 h. In the beginning, all emulsions presented a low content of peroxides (<5 meq hydroperoxide/kg emulsion), which did not differ (*p* > 0.05) among them. After 60 h, results showed that samples with higher values of primary oxidation products corresponded to the control (sample without HSL extract) and the emulsion with spheres of 50% ethanol (100EtOH). In these samples, PV increased according to the storage time, which corresponded to 236 ± 8.6 and 224 ± 20.4 meq. hydroperoxide/kg emulsion, respectively, at the end of the storage time. On the contrary, both emulsions that contained spheres of HSL extract (10HSL and 20HSL) showed the best oxidative stability. After ~154 h of storage, 10HSL and 20HSL samples remained stable against oxidation (Figure 10), although, from this point, these emulsions exhibited an exponential increase in their corresponding PV. No differences (*p* > 0.05) were observed between 10HSL and 20HSL samples after 240 h of storage (51.9 ± 0.6 and 52.0 ± 0.4 meq. hydoperoxides/kg emulsion, respectively). As observed in Figure 10, HSL spheres (20HSL and 10HSL) reduced the formation of hydroperoxides by ~80% if compared with the control emulsion (without spheres and 100EtOH).

**Figure 11:**
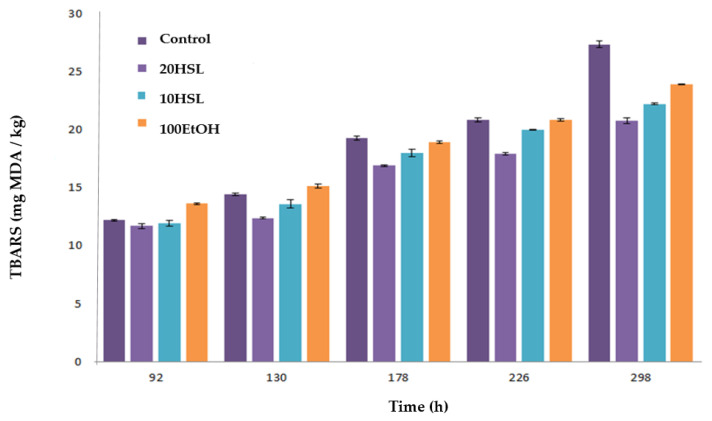
Evolution of thiobarbituric-acid-reactive substances (TBARS) of emulsions during their storage at 33 °C. Control: emulsion without anything; 100EtOH: emulsion with spheres of 50% ethanol; 10HSL: emulsions with 10 spheres of HSL extract, 20HSL: emulsions with 20 spheres of HSL extract. HSL: *Hibiscus sabdariffa* L. Emulsion: Tween-20 (1%, w/w), Milli-Q water (89%, w/w) and methyl linoleate (MeLo, 10%, w/w) oil. mg MDA/kg: mg of malondialdehyde/kg emulsion; error bars represent the standard deviation (n = 3).

Secondary oxidative products were monitored through TBARS assay at the initial time and after 96 h of the preparation of the samples. In the beginning, all samples presented similar contents of thiobarbituric-acid-reactive substances, although emulsions with spheres with HSL extract (10HSL and 20HSL) showed the lowest values. During the storage, the control exhibited a remarkable increase of TBARS, while these compounds showed a slower increase in emulsions with spheres (HSL or EtOH). After ~300 h of storage, 20HSL and 10HSL samples exhibited the highest inhibitory activity towards the formation of TBARS, followed by 10HSL > EtOH > Control (Figure 11). These results could also be attributed to the least hydroperoxide accumulation in 20HSL and 10HSL samples. Phenolic compounds contained in HSL extracts (such as chlorogenic acid, quercetin, and kaempferol) could interfere with the oxidative cycle of these emulsions [20,26]. These natural antioxidants probably played an important role in inhibiting the breakdown of hydroperoxides to TBARS, and supporting the previous findings concerning the potential antioxidant activity of the extracts of these plants against food systems [10,26]. Figure 11 shows that the formation of TBARS products was reduced by ~20% when HSL spheres were included in o/w emulsions if compared with the control.

The evolution of the pH value of emulsions with HSL spheres exhibited a similar and slight decrease during their storage time (from 2.57 ± 0.30 to 2.11 ± 0.09 in 10HSL and from 2.60 ± 0.13 to 2.06 ± 0.06 in 20HSL). On the contrary, a greater decrease in pH values of the control (from 2.02 ± 0.21 to 1.61 ± 0.16) and the emulsions with EtOH spheres (from 2.38 ± 0.20 to 1.76 ± 0.26) was observed, probably because of the new by-products generated as a consequence of the propagation of oxidation reactions. At lower pH values, the velocity of lipid oxidative reactions tends to accelerate, since the antioxidants could lose their effectiveness [27,28].

## 4. Conclusions

This study demonstrated the antioxidant capacity and scavenging activity of extracts of *Hibiscus Sabdariffa* L. (HSL) (with 50% ethanol) encapsulated in calcium alginate spheres. These spheres improved the oxidative stability of o/w emulsions (by reducing the produced substances of primary and secondary oxidation reactions), which directly depended on the number of HSL spheres included in these emulsions. The composition of the external medium was demonstrated to be an important factor to determine the physical stability of the spheres (diameter and weight) and also the release of the phenolic compounds contained in these alginate spheres. The scavenging capacity of simulants with spheres that contain HSL extracts was also demonstrated, which directly depends on the composition of the medium in which the spheres are dispersed. This work also indicated the potential of alginate spheres for encapsulating antioxidant compounds as a disruptive strategy of innovation for industrial applications. Nowadays, the encapsulation of bioactive compounds from plants is a potential tendency of industry in order to protect these substances through innovative delivery systems. This study sought to complement the current state of the art in order to overcome the above-mentioned challenges.

## Figures and Tables

**Table 1:** Regression kinetics of the evolution of total phenolic compound (TPC) content of the samples.

Simulants ^2^	TPC ^1^				
	Section 1		Section 2		Section 3
	Slope ^3^	Intercept	Slope	Intercept	Maximum Value [mg GAE/mL] ^4^
W	0.0206	0.0189	0.0026	0.0377	0.0560 ^c^
AA	0.0397	0.0206	0.0035	0.0587	0.0793 ^a^
50EtOH	0.0306	0.0195	0.0039	0.0467	0.0699 ^b^
100EtOH	0.0400	0.0149	0.0033	0.0507	0.0682 ^b^

^1^ TPC: Total phenolic compounds; ^2^ Simulants: W: water medium, AA: Acidified water with 3% acetic acid (v/v), 50EtOH: ethanol/water 50:50 (v/v), 100EtOH: pure ethanol; ^3^ Slope: mg GAE/mL × h; ^4^ Concentration: mg gallic acid equivalents (GAE) per mL of extract (w.s.). ^a–c^ Values with the same superscript did not present significant differences (*p* < 0.05).

**Table 2:** Regression kinetics of the evolution of Trolox equivalent antioxidant capacity (TEAC) of the samples.

Simulants ^2^	TEAC ^1^				
	Section 1		Section 2		Section 3
	Slope ^3^	Intercept	Slope ^3^	Intercept	Maximum Value [mmol Trolox/mL] ^4^
W	0.1794	0.1719	0.0129	0.3208	0.4177^a^
AA	0.0939	0.2062	0.0192	0.301	0.4370 ^a^
50EtOH	0.2057	0.1719	0.0109	0.3449	0.4222 ^a^
100EtOH	0.1582	0.1879	0.0117	0.3433	0.4164 ^a^

^1^ TEAC: Trolox equivalent antioxidant capacity; ^2^ Simulants = W: water medium, AA: acidified water with 3% acetic acid (v/v), 100EtOH: pure ethanol, 50EtOH: ethanol/water 50:50 (v/v); ^3^ Slope: mmol Trolox/mL × h; ^4^ Concentration: mmol of Trolox/mL of extract (w.s.). ^a^ Values with the same superscript did not present significant differences (*p* < 0.05).

**Table 3:** Regression kinetics of the evolution of Trolox equivalent antioxidant capacity (DPPH) of the samples.

Simulants ^2^	DPPH ^1^				
	Section 1		Section 2		Section 3
	Slope ^3^	Intercept	Slope ^3^	Intercept	Maximum Value [mmol Trolox/mL] ^4^
W	0.1057	0.2004	0.0233	0.2767	0.4268 ^b^
AA	0.1882	0.2943	0.0278	0.4418	0.6176 ^a^
50EtOH	0.1597	0.2635	0.0346	0.392	0.5936 ^a^
100EtOH	0.2475	0.1892	0.0274	0.4421	0.6050 ^a^

^1^ DPPH: 2,2-difenil-1-picrilhidracilo; ^2^ Simulants = W: water medium, AA: acidified water with 3% acetic acid (v/v), 100EtOH: pure ethanol, 50EtOH: ethanol/water 50:50 (v/v); ^3^ Slope: mmol Trolox/mL × h; ^4^ Concentration: mmol of Trolox/mL of extract (w.s.). ^a,b^ Values with the same superscript did not present significant differences (*p* < 0.05).

**Table 4:** Regression kinetics of the evolution of oxygen radical absorption capacity (ORAC) of the samples.

Simulants ^2^	ORAC ^1^				
	Section 1		Section 2		Section 3
	Slope ^3^	Intercept	Slope ^3^	Intercept	Maximum Value [mmol Trolox/mL] ^4^
W	6.3532	6.426	1.2067	12.083	19.6686 ^b^
50EtOH	6.0506	10.415	1.1493	15.803	23.0273 ^a^
100EtOH	6.0791	10.727	1.2912	16.221	23.8159 ^a^

^1^ ORAC: oxygen radical absorption capacity; ^2^ Simulants = W: water medium, AA: acidified water with 3% acetic acid (v/v), 100EtOH: pure ethanol, 50EtOH: ethanol/water 50:50 (v/v); ^3^ Slope: mmol Trolox/mL × h; ^4^ Concentration: mmol of Trolox/mL of extract (w.s.). ^a,b^ Values with the same superscript did not present significant differences (*p* < 0.05).

**Table 5:** Concentration of HSL extract and percentage of loss (from the interior to the simulant, in brackets) of chlorogenic acid, quercetin, and kaempferol in the spheres after 6 h of contact. The percentage was calculated taking into account the initial concentration (inside the sphere) and the final concentration (in the simulant) and the volume of simulant.

Reference ^1^	Concentration ^2^ (% Loss ^3^)		
	Chlorogenic Acid	Quercetin	Kaempferol
HSL extract	73.911	13.583	1.332
HSL in W	0.919 (37.6)	0.083 (69.2)	0.006 (75.6)
HSL in AA	1.049 (28.8)	0.109 (59.6)	0.010 (63.8)
HSL in EtOH:W	0.766 (48.0)	0.123 (54.7)	0.013 (51.7)
HSL in 100EtOH	0.291 (80.2)	0.116 (57.0)	0.011 (57.9)

^1^ W: water medium, AA: acidified water with 3% acetic acid (v/v), 100EtOH: pure ethanol, EtOH:W: ethanol/water 50:50 (v/v); HSL: *Hibiscus sabdariffa* L.; ^2^ Concentration: ppm.; ^3^ % loss: percentage of loss.

## Data Availability

The data presented in this study are available on request from the corresponding author.
